# Lipids for CD8^+^ TILs: Beneficial or harmful?

**DOI:** 10.3389/fimmu.2022.1020422

**Published:** 2022-10-07

**Authors:** Duojiao Wu, Yuwen Chen

**Affiliations:** Jinshan Hospital Center for Tumor Diagnosis & Therapy, Jinshan Hospital, Fudan University, Shanghai, China

**Keywords:** Exhausted T cell, cholesterol, fatty acid oxidation, CD8+ T cell, metabolism, tumor microenvironment (TME), fatty acid

## Abstract

Lipids and lipid metabolism play crucial roles in regulating T cell function and are tightly related to the establishment of immune memory. It is reported that tumor-infiltrating CD8^+^T lymphocytes (CD8^+^TILs) burn fats to restore their impaired effector function due to the lack of glucose. Conversely, fatty acids (FAs) and cholesterol in the tumor microenvironment (TME) drive the CD8^+^ TILs dysfunction. The origin of dysfunctional CD8^+^ TILs shares important features with memory T cell’s precursor, but whether lipids and/or lipid metabolism reprogramming directly influence the memory plasticity of dysfunctional CD8^+^ TILs remains elusive. It is necessary to understand the interplay between cellular lipid metabolism and dysfunction of CD8^+^ TILs in the case of targeting T cell’s metabolism to synergize cancer immunotherapy. Therefore, in this review, we summarize the latest research on CD8^+^ TILs lipid metabolism, evaluate the impacts of lipids in the TME to CD8^+^ TILs, and highlight the significance of promoting memory phenotype cell formation by targeting CD8^+^ T cells lipid metabolism to provide longer duration of cancer immunotherapy efficacy.

## Introduction

CD8^+^ T cells are the effector cells mediating anti-inflammatory and antitumor immunity, however, they become dysfunctional (a hyporesponsive state) due to prolonged exposure to tumor antigens. On the one hand, CD8^+^ T cells cannot be primed *via* inflammatory signals because of the nature of tumor antigens, which leads to an anergic cell state (1). On the other hand, nutrient deprivations and toxic metabolites accumulation in the TME together with the reprogramming of T cell metabolism induce CD8^+^ TILs exhaustion (2, 3). Cancer immunotherapy targeting T cells, such as immune checkpoint inhibitors (ICIs), restores the tumor-killing effect of CD8^+^ T cells by combating the negative immune regulators and has achieved breakthroughs in cancer treatment (4). However, ICIs cannot provide patients with durable therapeutic efficacies due to the exhaustion-related epigenetic reprogramming of CD8^+^ TILs (5). Thus, harnessing CD8^+^ T cells metabolism to improve cancer immunotherapy has emerged to be a novel therapeutic strategy.

Lipids as one of the most essential nutrients are the components of the cellular membrane and signal transducers. In different immune response microenvironments, lipids and lipid metabolic programs can promote or inhibit the viability and function of T cells (6). Effector (Teff) T cells upregulate lipid anabolism to meet their growing biomass molecules needs, while memory T (Tmem) cells burn fats to stay in a relatively quiescent and persistent state (7). Notably, there have been arguments about the impacts of lipids on CD8^+^ TILs. CD8^+^ TILs upregulate fatty acid oxidation (FAO) to remain tumor-killing effective (8), while lipids accumulated in the TME can directly drive CD8^+^ TILs exhaustion (9–12). Here, we compare the different opinions on cellular lipid metabolism and function of CD8^+^ TILs and discuss how to target CD8^+^ TILs lipid metabolic program to promote antitumor immunity and induce memory phenotypic CD8^+^ T cells to provide cancer immunotherapy durable therapeutic effects by utilizing lipid metabolism.

## Lipids and lipid metabolism regulate T cell activation and differentiation

T cell metabolism is reprogrammed downstream of TCR and CD28 mediated signaling after activation (13) and the FAs requirements of T cells vary among different functional phenotypes (**Table 1**). Consistent with the fast-dividing behavior, Teff cells upregulate the intake of extracellular FAs. After activation, the naive T (Tnaive) cells accumulate metabolites of the fatty acid synthesis (FAS) pathway (23). Inhibiting the activity of phosphatidylinositol-3-kinase (PI3K) and Mammalian target of rapamycin (mTOR), the enrichment of Sterol regulatory element binding proteins (SREBPs) at the gene promotors of lipid synthesis enzymes was declined (16). Similarly, impaired mTOR complex 1 (mTORC1) activity led to a decrease of T cell receptor (TCR) and CD28 mediating *de novo* lipid synthesis in antigen-specific T cells (24). In contrast, CD8^+^ T cells cultured *in vitro*, using Akt or mitogen-activated protein kinase (MAPK) kinase (MEK) inhibitors, were found to have increased FAO and differentiated into memory cells, revealing a lipid catabolism status opposite to Teff cells (25, 26). These findings suggest that PI3K/Akt and MAPK pathways imprint cellular differentiation fates through reprogramming lipid metabolism, which makes sense since Tmem cells start to develop after Teff cells contract. Interestingly, another study found that the CD28 costimulatory signal also reprogramed the mitochondrial respiratory capacity at the early stage of T cell activation and contributed to immune memory formation (27). With enhanced mitochondrial carnitine palmitoyltransferase I A (CPT1A) expression, the Tmem cells manifest increased FAO without increasing extracellular FAs uptake. Instead of directly utilizing extracellular FAs for FAO, Tmem cells firstly synthesize their own FAs from extracellular glucose and store them in the endoplasmic reticulum by converting them into triacylglycerol (TAG), then the TAG is hydrolyzed by lysosomal acid lipase (LAL) to provide FAO pathway free FAs (18). In addition, interleukin (IL) -7 upregulates the glycerol transporter aquaporin 9 (AQP9) expression of Tmem cells to increase transport of extracellular glycerol for TAG synthesis, which is important in maintaining Tmem cells homeostasis (28). This seemingly less effective unique lipid metabolic program, named “store and burn” model (28), not only reflects the fact that Tmem cells denote a relatively quiescent cell state (low anabolism level) and do not rely on exogenous fatty acids, but also prevent Tmem cells from potential lipotoxicity by storing free FAs in the form of TAG. Notably, short chain FAs (SCFAs) derived from microbiota promote the antigen-activated CD8^+^ T cells memory potential by switching the cellular metabolism to OXPHOS and FAO from glycolysis (20). In addition, SCFAs also promote Th1 and Th17 subsets differentiation through epigenetic modifications (29). In conclusion, the heterogeneity of lipid metabolism is integrated in the immunological signaling, while lipids in the immune microenvironment can also rewire cellular metabolic programs and gene expressions to selectively dictate cellular differentiation fates.

**Table 1:** lipids/lipid metabolites and lipid metabolism profile in CD8^+^ T cell subsets.

Cell type	Lipids	Lipid source	Lipid metabolic phenotype	Effect on T cell	Mechanism	Reference
**Tnaive cell**	FAs	Extracellular uptake	OXPHOS/FAO	Fuels for quiescent metabolic needs	Tonic TCR signaling and inhibition by Treg cells?	(14, 15)
**Teff cell**	FAs	Intracellular biosynthesis	FAS	Celluar building blocks for rapid proliferation	PI3K/Akt/mTOR signaling	(16)
	Membrane cholesterol	Intracellular biosynthesis	Reduced cholesterol esterification	Enhanced tumor-killing cytotoxicity	Immunological synapse formation	(17)
**Tmem cell**	FAs	Intracellular biosynthesis	“store and burn” mode of FAO	Increased mitochondrial reserve prepared for rapid energy needs	IL-7/AQP9/TAG axis	(7, 18)
		Extracellular uptake	FAO	Induction of memory differentiation	H3K27ac modification of pro-memory genes	(19)
	SCFAs	Microbiota	OXPHOS/FAO	Fuels for long-term survival	GPR41 and GPR43 mediated SCFAs sensing	(20)
**Tex cell**	LCFAs and VLCFAs	TME	Impaired FAO	Impaired tumor-killing cytotoxicity	Lipotoxicity	(9)
	AAs and ox-LDLs				Lipid peroxidation and ferroptosis	(10, 11)
	Cholesterol	TME	Cholesterol overload	Induction of exhaustion	XBP1-mediated transcription of PD-1 and 2B4	(12)
	Oxysterol	TME	Cholesterol oxidation	Induction of Tc9 cell exhaustion	LXR sumoylation	(21)
**Trm cell**	FAs	TME	FAO	Fuels for long-term survival	FABP4/FABP5 mediated FAs uptake	(22)

## Lipids differentially dictate CD8^+^ TILs function

### Fatty acids

**Figure 1:**
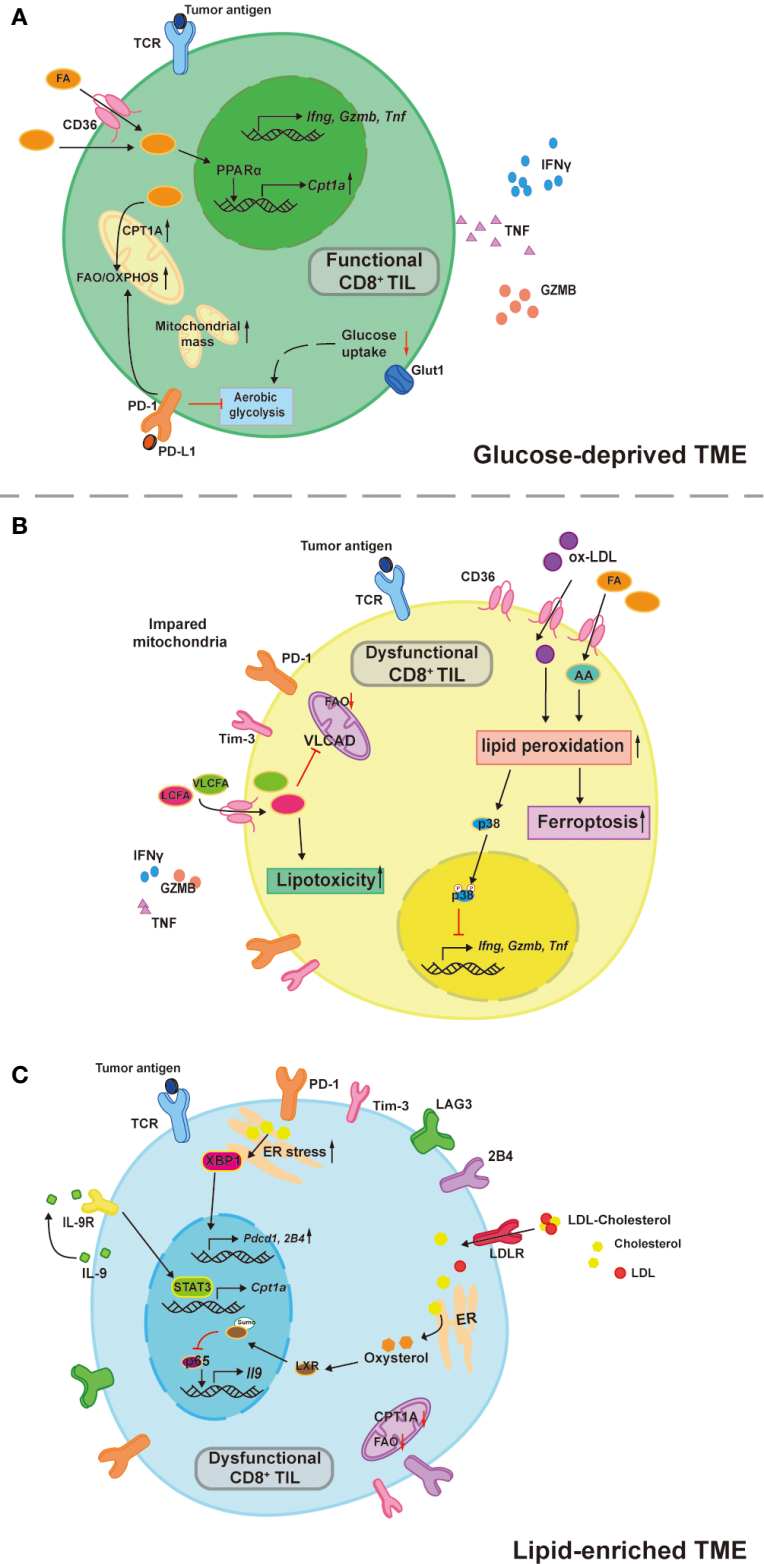
TME lipids dictate CD8^+^ TILs functional heterogeneity. **(A)** CD8^+^ TILs utilize aerobic glycolysis to support their antitumor effects, which is inhibited in a glucose-deprived TME. Instead of relying on glucose, CD8 +TILs upregulate PPARα-mediated CPT1A expression in response to the environmental FAs and the FAO is therefore increased to compensate the energy deficit. This compensatory circuit is further reinforced by PD-1 signaling due to its inhibitory effects on aerobic glycolysis. **(B)** Increased uptake of TME FAs and oxidized lipids by CD36 drive CD8^+^ TILs dysfunction. Both Arachnoid acid (AA) and oxidized low density lipoprotein (ox-LDL) induce abnormal cell death by ferroptosis, ox-LDL also increases p38 phosphorylation, which inhibits the transcription of effector cytokines like TNFα and IFNγ. LCFAs and VLCFAs poison CD8^+^ TILs through lipotoxicity and inhibit the VLCAD expression that in turn accelerates the accumulation of LCFAs and VLCFAs. **(C)** Tc9 cells are resistant to lipid peroxidation and ferroptosis by its IL-9 autocrine-mediated STAT3/FAO pathway, which is impaired in the cholesterol-enriched TME. Oxysterol promotes the sumoylation of LXR that inhibits the p65 binding on IL-9 promoter and therefore decreased expression. Moreover, cholesterol accumulation in the cytoplasm promote ER stress sensor (XBP1) expression to directly induce inhibitory receptor expressions including PD-1 and 2B4.

CD8^+^ TILs can utilize FAs as an alternative source of energy fuels in glucose-deprived TME to remain robust, but excessive FAs accumulated in the TME is what causes the CD8^+^ TILs dysfunction. Zhang et al. reported that CD8^+^ TILs upregulated FAO by Peroxisome proliferator-activated receptor alpha (PPARα) signaling to maintain effector function when entering hypoxic and hypoglycemic TME (8) (**Figure 1A**). Conversely, another group of researchers presented that in the TME of Pancreatic ductal adenocarcinoma (PDA) containing high long chain fatty acids (LCFAs), excessive intracellular LCFAs induced CD8^+^ TILs dysfunction (9) (**Figure 1B**). With tumor progression, a steady buildup of TME FAs is an environmental cue to the PPARα-mediating FAO upregulation (8), resulting in the increased fatty acid translocase or CD36 expression on the CD8^+^ TILs for adaptation to lipid-enriched TME (10, 11). In addition, impacted by the Programmed Cell Death protein 1 (PD-1) signaling, T cells’ aerobic glycolysis in the early phase of activation is restricted and T cells shift their metabolic program towards FAO for energy production with enhanced CPT1A expression (30). PD-1^+^CD8^+^ TILs possess superior antitumor effects and lipids uptake (31). However, increased uptake of extracellular FAs and oxidized low density lipoproteins (ox-LDLs) through CD36 causes lipid peroxidation and ferroptosis, which impairs the effector cytokines production including tumor necrotic factor (TNF) and interferon-gamma (IFN-γ), finally leading to T cell exhaustion (10, 11) (**Figure 1B**). The ox-LDLs promote p38 phosphorylation and lipid peroxidation in a CD36 dependent manner. The increased p38 phosphorylation is responsible for the decreased transcription of effector cytokines genes and can be rescued by glutathione peroxidase 4 (GPX4) inhibition of lipid peroxidation (11). Likewise, arachidonic acid (AA), a poly-unsaturated fatty acid, is responsible for the lipid peroxidation and ferroptosis (10). LCFAs accumulated in the cytoplasm can directly damage CD8^+^ T cell’s mitochondria, while the downregulated very-long-chain acyl-CoA dehydrogenase (VLCAD) further accelerates LCFAs and very long chain fatty acids (VLCFAs) accumulation causing lipotoxicity to impair CD8^+^ T cells function (9). Therefore, the influence of FAs on CD8^+^ TILs depends on their intracellular concentrations, chemical forms, and how they are metabolized or processed. CD8^+^ TILs reprogram their metabolic program to utilize TME FAs to overcome the shortage of environmental glucose, but TME FAs and oxidized lipids start to accumulate with tumor progression and poison CD8^+^ TILs *via* lipid peroxidation, ferroptosis, and lipotoxicity.

It is crucial to understand how to instruct CD8^+^ TILs consistently and stably metabolize TME FAs without being influenced by those unwanted adverse effects that cause T cell dysfunction, particularly when the TME is glucose-deprived and/or lipid-enriched. Promoting CD8^+^ T cell’s mitochondrial biogenesis and thus improved mitochondrial fitness is the prerequisite for T cells to consistently catabolize LCFAs since the CPT1A is the rate-limiting enzyme that catalyzes LCFAs oxidation and mitochondrial dysfunction is one of the metabolic characteristics of exhausted CD8^+^ T (Tex) cells (2). Indeed, PPARα agonists work by increasing mitochondrial CPT1A expression to enhance CD8^+^ T cell’s FAO (32, 33). Pharmacologically boosting CD8^+^ T cell’s mitochondrial biogenesis by Peroxisome proliferator-activated receptor gamma coactivator 1-alpha (PGC-1α) activators (bezafibrate and oltipraz) was proven to be synergetic in assisting anti-PD-1 therapy in mice (34). The PGC-1α together with its cofactors induce a series of transcription factors that further activate FAO and oxidative phosphorylation (OXPHOS), which promote mitochondrial biogenesis and CD8^+^ T cell proliferation. In line with this, enforced PGC-1α expression in CD8^+^ T cells was shown to be effective in maintaining mitochondrial and metabolic fitness, which resulted in longer CD8^+^ TILs persistence in the harsh TME and better tumor suppression in the mouse melanoma model due to the central memory T cells (Tcm) formation (35). These studies suggest that mitochondrial activators seem to sensitize cancer patients to PD-1 blockade therapy and generate long-term antitumor immunity (**Table 2**). Interestingly, another group of researchers found that the PD-L1 inhibitor favored tissue resident memory T (Trm) cells’ lipid metabolism from FAs competition with cancer cells in the TME and unleashed their anti-tumor functions in gastric adenocarcinoma (22). This suggests that certain agents interfering with the immune checkpoint signals can also maintain the lipid metabolic fitness of memory cells to support their long-term survival in harsh TME. While in the context of immunosuppressive TME, it seems more sensible to target CD36 for restricting FAs uptake of CD8^+^ TILs. By doing so, CD8^+^ TILs had lower ferroptosis mediated by CD36 and acquired improved antitumor effects, especially when combined with anti-PD-1 therapy compared with the control counterpart (10). In contrast to CD8^+^ TILs, tumor-infiltrating CD4 regulatory T (Treg) cells with upregulated CD36 expression persisted in the TME and suppressed the antitumor immunity (37). This converse effect was found to be the result of CD36-Peroxisome proliferator-activated receptor beta (PPAR-β) regulated mitochondrial dynamics and metabolic adaptation. Intriguingly, both pharmacologically and genetically targeted CD36 of Treg cells specifically induced their intratumoral apoptosis, leading to increased CD8^+^ T cells infiltration in the tumor and enhanced anti-PD-1 therapeutic effects (37). High expressions of CD36 were also reported in other immunosuppressive cells, such as tumor-associated macrophages and myeloid-derived suppressor cells, and the malignancies of various human cancer types (38). Moreover, CD36 neutralizing antibodies (FA6.152 and JC63.1) were previously shown to be effective in treating mouse metastatic tumors (39). Thus, therapies targeting CD36 may produce a comprehensive efficacy that works by acting on various cell types in the TME, which needs to be confirmed by future studies (**Table 2**). In addition, there is a specific subset of CD8^+^ TILs named cytotoxic T lymphocyte subset 9 (Tc9) is resistant to tumor derived ROS induced lipid peroxidation and ferroptosis (43), which could be utilized for adoptive cell therapy (ACT) in treating cancer (44).Xiao et al. reported that Tc9 cells attained longevity in TME through IL-9/STAT3/FAO pathway that avoid them from lipid peroxidation and ferroptosis (43) (**Figure 1C**). However, this seemingly lipid resistant ACT candidate was found to become dysfunctional in cholesterol enriched TME (discussed below) (21). In conclusion, maintaining mitochondrial fitness is the key for CD8^+^ TILs to utilize TME fatty acids and persist in lipid-enriched or glucose-deprived TME. Restricting fatty acids intake of CD8^+^ TILs combined with adoptively transferring lipid-peroxidation-resistant CD8^+^ T cells may be more efficient in combating immunosuppressive TME.

**Table 2:** Targeting lipid metabolism for cancer immunotherapy.

Therapy	Molecule	Target	Impact on cellular metabolism	Combinatorial regimen	Mechanism of therapeutic effect	Cancer type	Reference
**Targeting mitochondria**	Bezafibrate	PGC-1α	Increased FAO	anti PD-L1 mAb	Sensitized PD-1 blockade, Tcm cells formation	MC38 colorectal carcinoma and B16-Ova melanoma models	(33–35)
	Oltipraz						
	GW501516	PPARα and PPARδ/β	Increased CPT1a-mediated FAO	ACT	Enhanced transferred therapeutic CD8^+^ T cells persistence	B16 melanoma model	(32)
	MPC inhibitors	MPC	Increased FAO and OXPHOS	anti-CD19 CAR T cell	Increased H3K27ac modification of pro-memory genes	Nalm6 cells induced human leukemia model	(36)
**Targeting fatty acid translocase**	anti CD36 mAb, FA6.152 and JC63.1	CD36	Restriction of extracellular FAs uptake	anti PD-1 mAb	Decreased CD8^+^ TILs ferroptosis and lipid peroxidation, impaired intratumoral CD4^+^ Treg, inhibition of other immunosuppressive cells like MDSCs and TAMs, suppression of metastatic tumor cells	B16 melanoma and YUMM1.7 melanoma models	(10, 37–39)
**Targeting Cholesterol metabolism**	Avasimibe	ACAT1	Decreased Cholesterol esterification	anti PD-1 mAb	Increased effector cytokines, proliferation, TCR clustering and immune synapse formation by CD8^+^ TILs	B16 melanoma model	(39)
				Kras peptide cancer vaccine	Improved CD8^+^ Teff/CD4+ Treg ratio in tumor	KrasLA1 murine lung cancer model	(40)
	Simvastatin	HMGCR	Inhibition of mevalonate biosynthesis	Cancer vaccine and/or anti PD-1 mAb	Reduced small-GTPase geranylgeranylation, enhanced antigen presentation for T cell activation	B16-Ova melanoma	(41)
**Targeting immunological signal transduction**	AKT inhibitors	AKT	Increased FAO and mitochondrial SRC	ACT	Increased memory-like CD8^+^ T cells formation	B16 melanoma model	(25)
			Inhibited glycolysis	anti-CD19 CAR T cell	Prolonged anti-CD36 CAR T cells persistence after adoptive transfer	NALM6 acute lymphoblastic leukemia (ALL) model	(42)
	MEK inhibitors	MEK	Increased FAO and mitochondrial SRC	ACT	Increased CD8^+^ TSCM formation	B16 melanoma and TC-1 cell tumor models	(26)

### Cholesterol

Cholesterol is another common lipid species consumed by fast-growing cells including cancer cells and Teff cells. Like FAs, the roles of cholesterol in T cell functions are controversial. Lowering cellular cholesterol level helps restore CD8 TILs effector function (12, 21, 45), while that may inhibit TCR signaling at the early phase of CD8 T cells activation (16, 17). Within cholesterol-enriched TME, cellular cholesterol positively correlates with the PD-1, 2B4, LAG-3, and Tim3 expression on CD8^+^ TILs. Accumulated cholesterol sensed by endoplasmic reticulum stress sensor, X-box binding protein 1 (XBP1), consequently increases PD-1 and 2B4 transcriptions, promoting CD8^+^ TILs exhaustion (12) (**Figure 1C**). In addition, oxysterol, a cholesterol metabolite, inhibits p65 binding on *Il9* promotor through liver X receptors (LXRs) sumoylation, leading to Tc9 cell dysfunction (21) (**Figure 1C**). One recently published article identified genetic ablation of Fibp led to better survival and superior antitumor effector function of human and mice CD8^+^ TILs in high cholesterol TME. Fibp knockout CD8^+^ TILs reduced extracellular cholesterol uptake, downregulated cholesterol biosynthetic enzymes, and rendered lower cellular cholesterol levels compared to control counterparts, which was independent of extracellular cholesterol doses (45). By contrast, in an earlier study, researchers found that restricting CD8^+^ T cells’ cholesterol esterification was beneficial for their antitumor function. ACAT1 is a key enzyme that catalyzes cholesterol esterification, when inhibited by avasimibe, the CD8^+^ T cells membrane cholesterol levels were elevated, which resulted in the enhanced antitumor effect by facilitating immunological synapse formation and antigen recognition (17). Despite the cholesterol biosynthetic enzymes mRNA expressions being upregulated in the ACAT1 deficient CD8^+^ T Cells, it was unclear whether the resultant better antitumor effect was established in a cholesterol-enriched microenvironment. Interestingly, ACAT1 inhibitor (avasimibe) monotherapy did enhance CD8^+^ TILs effector functions regardless of PD-1 expression levels and higher survival rates were observed in mouse melanoma combining anti-PD-1 mAb, suggesting that ACAT1 inhibitor might be a quality adjuvant for anti-PD-1 immunotherapy (17) (**Table 2**). The immunological synapse is regarded as a lipid raft structure and membrane cholesterol contents directly affect lipid raft and membrane dynamics (46). Furthermore, cholesterol was found to affect TCR-CD3 molecular configuration, inhibit TCR signal transduction, and may play a role in preventing T cells over activation (47) (**Figure 1C**). Taken together, unlike FAs, cholesterol regulates T cell functions through its intracellular concentration and subcellular location.

Drugs targeting cholesterol metabolism are widely applied in clinical practice, managing cholesterol metabolic disorders and have adjuvant effects in prolonging cancer patients’ survival (48). More importantly, scientific research has gained new insights into their potential clinical applications in cancer immunotherapy. Statins, HMGCR inhibitors, targeting the cholesterol biosynthetic pathway were reported to extend the life spans of cancer patients (48). However, according to the most recent study, it is unfavorable to directly target CD8^+^ T cells’ cholesterol biosynthesis by statins. Statins elevated the cellular cholesterol levels in activated CD8^+^ T cells and inhibited their proliferation (45). Intriguingly, in an earlier study, simvastatin, a lipophilic statin, was found to not only act as a cancer vaccine adjuvant but also synergize anti PD-1 therapy in multiple animal models through decreasing the geranylgeranylation of small GTPases and regulating endosomal trafficking in dendritic cells which led to better antigen presentations (41) (**Table 2**). Opposite to directly targeting the cholesterol biosynthetic enzyme, genetically targeting *Fibp* generated favorable cellular cholesterol-lowering effects in CD8^+^ T cells while remained their basal cholesterol biosynthesis (45). However, the biological effects of *Fibp* still remain largely unknown, and the beneficial effect in sustaining CD8^+^ T cells resilience in cholesterol enriched TME waits for further investigations to expand its potential clinical applications in cancer immunotherapy. Since accumulated cholesterol in the TME is generated from cancer cells and stromal cells (49, 50), future studies should evaluate the feasibility to target the cholesterol metabolism of cancer cells or stromal cells for cancer therapy rather than CD8^+^ TILs because of the unfavorable effects of statins on activated CD8^+^ T cells. As discussed above, avasimibe promotes CD8^+^ TILs antitumor function through targeting the cholesterol esterification enzyme ACAT1, resulting in increased membrane cholesterol content and probably increased intracellular cholesterol level, but whether it is effective in a cholesterol enriched TME remains unclear (17). Another study working on the Kras peptide cancer vaccine found that avasimibe enhanced the tumor vaccine targeting Kras mutation in a murine lung cancer model (40). Avasimibe inhibited the cholesterol esterification of CD8^+^ TILs rather than cancer cells and reversed the ratio of CD8^+^/CD4 T cells, especially decreased CD4^+^CD25^+^FOXP3^+^ Treg cells infiltration in the TME. In conclusion, currently available drugs regulating cholesterol metabolism in clinical practice can improve antitumor immunity in mouse models either by directly targeting CD8^+^ TILs cholesterol metabolism or enhancing cancer vaccine efficacy.

Instructing the rational use of lipids by CD8+ TILs in the different metabolic microenvironments is the key to ensuring their optimal anti-tumor effects. As described above, growing evidence from the CD8^+^ T Cell lipid metabolism studies has pointed out several approaches targeting lipid metabolism possessing potential clinical use in (combination with) cancer immunotherapy (**Table 2**). For example, improving mitochondrial fitness is essential to keep cellular lipid metabolism intact; restricting FAs uptake has comprehensive therapeutic effects by acting on multiple immune cell types and tumor cells; appropriate timing of suppressing cholesterol biosynthesis yields better ICIs outcomes. Notably, clinical studies are needed in the future since most of these opinions are gained from animal studies. Understanding the TME lipid compositions and systematic lipid metabolism profiles of cancer patients will instruct researchers on how to target CD8^+^ TILs’ lipid metabolism for cancer immunotherapy.

## Lipid metabolism and memory development

**Figure 2:**
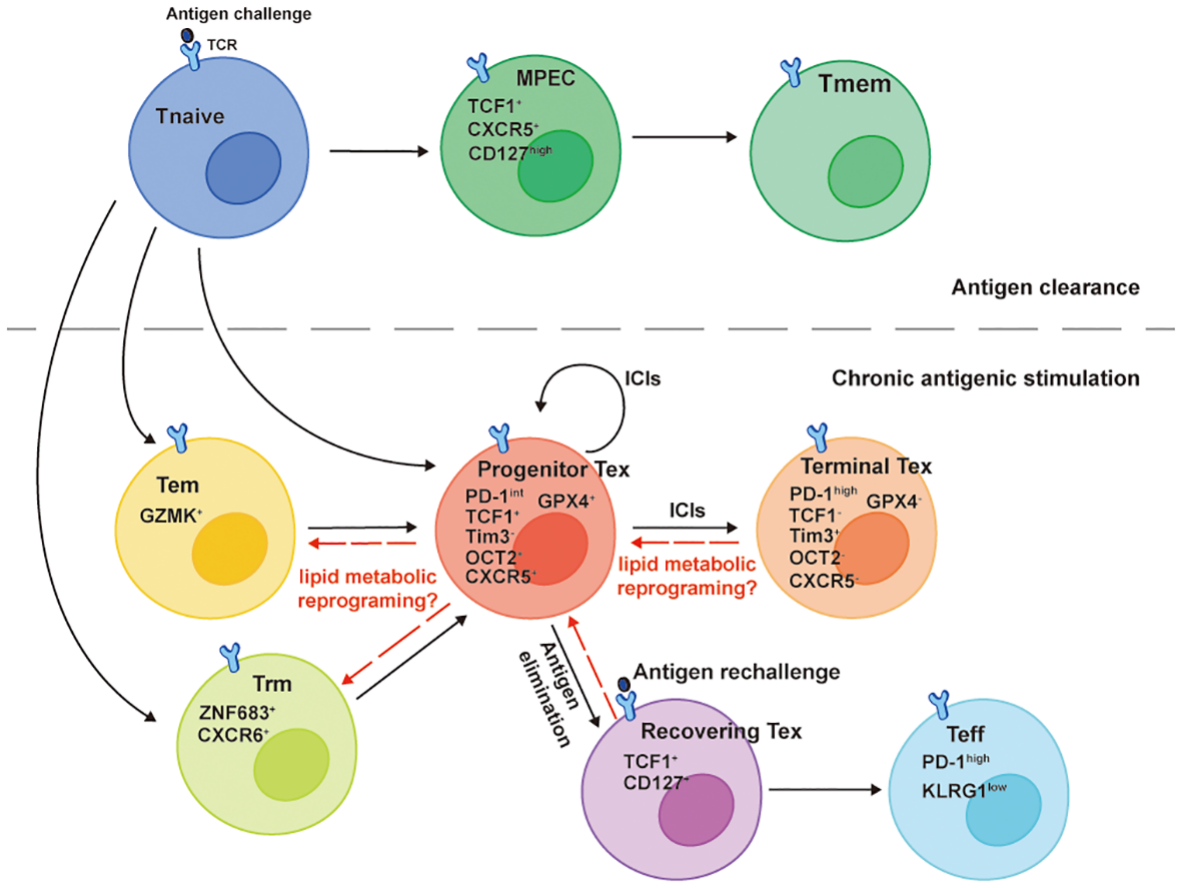
Developmental trajectories of Tex cells. The TCF1^+^Tim-3^-^ progenitor Tex cells denote a cluster of Tex cells with self-renew ability that differentiate into TCF1^-^Tim-3^+^ Terminal Tex cells when responding to ICIs, such as PD-1 inhibitors. TCF1 and newly identified OCT2 are signature molecules for the progenitor Tex cells memory-like plasticity. They emerge and develop from either GZMK^+^ Tem cells or ZNF683^+^CXCR6^+^ Trm cells in the chronic antigenic stimulation circumstance and share memory-like features with TCF1^+^CXCR5^+^CD127^high^ MPECs that give rise to Tmem cells after the antigen clearance. The TCF1^-^Tim-3^+^ terminal Tex cells lose lipid metabolic plasticity along with the downregulation of GPX4. Though they derive a fraction of recovering Tex cells reacquiring TCF1 and CD127 expressions after chronic antigen elimination, the PD-1^high^KLRG1^low^ Teff cells generated from the recovering Tex cells can’t efficiently eliminate a secondary antigen challenge because of the defect polyfunctionality.

It is well known that the Tmem cells provide hosts long-term immunity after Teff cells contract in preventing second infections or tumor recurrence. A pan-cancer study analyzing Tumor-infiltrated T cells at a single-cell resolution identified patients with low Tex cells and high Trm cells infiltrations had longer survival and higher response rates to PD-1 inhibitors (51). More importantly, developmental trajectory analysis through computational calculations revealed links between Tmem cells (Trm cells and Tem cells) and Tex cells (**Figure 2**). This suggests that preventing memory phenotypic CD8^+^ TILs from exhaustion is a potential strategy to assist PD-1 inhibitors or other ICIs. Since the overload of lipids in the TME can drive CD8^+^ TILs dysfunctional or exhausted, is it possible to target progenitor exhausted CD8^+^ TILs lipid metabolism for generating memory phenotypes (**Figure 2**)?

The Tex cell is a specific T cell differentiation state that has been widely reported in chronic infections and tumors, which is transcriptionally and epigenetically different from Teff cells and Tmem cells (52). However, studies in the past few years showed the origin of Tex cells shared similar features with Tmem precursor cells (**Figure 2**). A stem-cell like progenitor Tex cell subset (TCF1^+^Tim3^-^CD8^+^ T cell) proliferates, self-renews, and differentiates into terminal Tex cells (TCF1^-^ Tim3^+^CD8^+^ T cell) when responds to PD-1 inhibitors (53–55). A recent meta-analysis of CD8^+^ T cells epigenetics in chronic infections and tumors using Single-cell ATAC sequencing and RNA sequencing reported a common dysfunctional CD8^+^ T cell differentiation population expressing TCF1 emerged as early as day 7 in chronic antigen stimulation. In addition, this progenitor-like population shared the expression of the same genes with memory precursor effector cell (MPEC) in acute infection of LCMV mouse model, including *Tcf7, Cxcl10, Xcl1, Slamf6, Id3, Ccr7, Cxcr5, Tox* and *Ikzf2*, revealing an early divergent of CD8^+^ T cell differentiation fates in different immune responses (56). Despite (progenitor exhausted and dysfunctional CD8^+^ T cells) possessing memory-like capacities at the early time point of chronic antigen stimulations, terminally exhausted CD8^+^ T cells fail to completely regain the memory-like capacities after the elimination of antigens (57). After the clearance of chronic antigen stimulation, some Tex cells (named recovering Tex cells) reacquire TCF-1 and CD127 expressions and differentiate into Teff cells upon antigen rechallenge (**Figure 2**). However, the Teff cells derived from recovering Tex cells are less efficient in mediating secondary immune response, characterized by lower KLRG1, higher PD-1 expressions, and lack of polyfunctionality compared to those derived from Tmem cells (57). Taken together, CD8^+^ T cells acquire dysfunctional differentiation fates in the early phase of chronic antigen stimulations and proceed to a terminal exhausted state with distinct transcriptional and epigenetic reprogramming, losing memory plasticity (57–59).

The CD8^+^ TILs terminal exhausted differentiation state accompanies the decline of lipid metabolic plasticity. Although CD8^+^ TILs are committed to dysfunctional fates at the early stage of chronic antigen stimulations (56), they are still capable of sensing lipid metabolic stress and reprograming their metabolic programs towards OXPHOS (8). This suggests that like memory plasticity, the metabolic plasticity of progenitor exhausted CD8^+^ T cells is compatible with their transcriptional programs and epigenetic landscape, that is, the chromatins of genes regulating lipid metabolism are in open states. While antigen persists and terminal exhaustion develops, TCF1^-^Tim-3^+^CD8^+^ TILs upregulate CD36 with increasing oxidized lipids intake and downregulate glutathione peroxidase 4 (GPX4) (**Figure 2**), resulting in impaired anti-lipid peroxidation ability (11, 60). Similarly, in advanced PDA patients, CD8^+^ TILs accumulate certain LCFAs with decreased ACADVL (encoding VLCAD) transcription, accelerating the lipotoxicity mediated by LCFAs and VLCFAs (9). In other words, the downregulations of these lipid metabolic genes likely accompany the transcriptional and epigenetic reprogramming of Tex cells. In addition, lipids can directly interact with epigenetic modifying enzymes or their substrates to regulate T cell differentiation and functions. For example, SCFAs inhibit histone deacetylases (HDACs) leading to increased acetylation of S6K, which further promote Th1 and Th17 differentiations (29). Increased H3K9ac of HRE mediated by butyrate also contributes to increased production of IL-22 by CD4 T lymphocytes through promoting HIF1α binding on *Il22* promoter, which is important in establishing intestinal immunity (61). Similarly, increased FAO-generated acetyl-CoA can enforce the CD8^+^ T cells memory signatures including *Sell, Tcf7, Ccr7* through H3K37ac modification (19). Accumulated cholesterol in the TME enhances the transcriptional factor XBP1 binding on *the Pdcd1* promoter leading to increased CD8^+^ TILs PD-1 expression and exhaustion (12). However, whether lipids in the TME can regulate CD8^+^ TILs memory signatures and/or exhaustion signatures through epigenetic modifications hasn’t been examined and remain elusive. Future CD8^+^ TILs studies should be aimed at delineating the relationship between lipid metabolism and cell differentiation state-specific modifications from the perspective of epigenetics.

*In vitro* culture inducing memory phenotypic CD8^+^ T lymphocytes is the key to obtain long-term ACT and CAR T cell therapeutic effects (62), and most of them were achieved by promoting cellular FAO and mitochondrial fitness (**Table 2**). As mentioned above, mitochondrial activators help inducing Tcm cells formation and sensitize PD-1 blockade immunotherapy (34, 35) by potentiating mitochondrial OXPHOS and FAO. One recent study further highlighted the role of imprinting memory CD8^+^ T cells by increased FAO in facilitating CAR T cell immunotherapy (19). The Inhibition of mitochondrial pyruvate carrier (MPC) rewired the tricarboxylic acid cycle by enhanced FAO and glutamine oxidation instead of pyruvate, generating more acetyl-CoA for H3K27ac modifications of pro-memory genes. More importantly, this *in vitro* priming of CAR T cells by MPC inhibition works downstream of the glycolysis pathway, which avoids the inhibition of CAR T yield caused by interfered glycolysis (36), providing an alternative way to metabolically modify CAR T cells for long-lasting therapeutic effects (19). Other molecules targeting the PI3K/Akt and MAPK pathways were also found to be potential therapeutic strategies in similar ways. Though drugs targeting PI3K/Akt pathways have been being investigated for cancer monotherapy, the comprehensive effects on both cancer cells and antitumor T cells remain elusive. Intriguingly, CD8^+^ TILs treated with Akt inhibitors highly expressed CD62L and other memory-like surface markers, and the cellular mitochondrial spare respiratory capacity (SRC) and FAO were enhanced. After adoptive transfer into tumor-bearing mice, the volume of melanoma in mice decreased and the survival time prolonged (25). Akt inhibitors also achieved similar results in the model of anti-CD19 CAR-T cell therapy of B cell acute lymphoblastic leukemia (42). Likewise, MEK inhibitor (MEKi) can not only prevents CD8^+^ TILs from exhaustion, but also promotes FAO by enhancing mitochondrial biogenesis, and inducing the formation of stem cell like memory T cells (TSCM), providing a lasting antitumor effect for ACT (26). Indeed, the ERK2 MAPK pathway is indispensable for CD8^+^ T cell survival after TCR activation (63), and MEKi represses the priming of Tnaive cells and the expansion of CD8^+^ TILs in the CT26-tumor-bearing mice, which was thought to impair CD8^+^ TILs antitumor effects (64). However, MEKi potentiated the PD-L1 inhibitors’ therapeutic effects in combination therapy, which was attributed to the decreased CD8TILs exhaustive apoptosis regulated by TCR/MAPK/Nur77 axis in the TME (64). This suggests that the timing of MEKi administration is important in therapeutic regimens and highlights their translational effect in ACT applications since it’s easier to directly administer the drugs after the priming of naïve cell is finished *in vitro*. Though monotherapy of FDA-approved MEK inhibitors in cancer has been applied in the clinical treatment of several cancer types, patients acquired resistance due to the increased compensatory phosphorylated MEK were also reported (65). Hong et al. reported a novel MEKi-based regimen using a type II RAF inhibitor (RAFi) with an allosteric MEKi has tremendous improvement in suppressing the acquired MEKi resistance among cancers including KRAS, NRAS, NF1, BRAF non-V600, and BRAF V600 mutations through sequestering MEK from ERK. More importantly, this regimen systematically increased the CD8^+^ Tcm/CD4^+^Treg cells ratio and enhanced the intratumoral CD8^+^ Tem cells infiltration, reflecting a robust antitumor immunity (66). Therefore, drugs targeting the PI3K/Akt and MAPK pathways now appear to be promising candidates for cancer immunotherapy with intrinsic antitumor effect on cancer cells, and more studies are needed to further unravel the role of these cellular pathways in different immune microenvironments.

Taken together, lipids and lipid metabolism reprogramming may contribute to the loss of CD8^+^ TILs memory plasticity. Together with mitochondrial fitness, they are valuable targets for inducing memory cell formation in establishing lasting cancer immunotherapy including ICIs and ACT. These memory-like therapeutic cells have an enhanced ability to metabolize lipids, less dependence on aerobic glycolysis, and are not prone to exhaustion when entering glucose-deficient or lipid-enriched TME. Since the therapeutic cells of ACT are derived from autologous tumor-reactive TILs, it’s interesting to examine whether the progenitor exhausted Tcf1^+^Tim3^-^CD8^+^ T cells can be reprogrammed towards a memory-like phenotype through targeting lipid metabolism and/or mitochondrial fitness (**Figure 2**).

## Conclusion

The controversial roles of lipids and lipid metabolism have broadened our perspectives on the relations between environmental nutrients and CD8^+^ T cell heterogeneity. After activation, T cells upregulate the *de novo* fatty acid synthesis pathway and increase the uptake of exogenous fatty acids, supporting the biosynthesis of macromolecules for rapid cell expansion. On the contrary, during the Tmem cells differentiation, the cellular metabolic preference gradually shifts from aerobic glycolysis and lipid anabolism towards lipid catabolism, supporting a relatively quiescent cell state. TME is a nutrient-deprived and lipids-enriched microenvironment. Due to the metabolic stress, CD8^+^ TILs depend on OXPHOS for energy production and FAO increased. However, the increased FAs or lipids uptake also bring negative side effects for CD8^+^ TILs that lead to dysfunction or exhaustion, characterized by lipotoxicity, lipid peroxidation, and ferroptosis. Similarly, membrane cholesterol is crucial in transducing immunological signals, but the accumulated TME cholesterol can poison the CD8^+^ TILs inducing their exhaustion.

In the past decade, immunometabolism studies have revealed the interplay between cellular metabolism and immune functions, raising a consensus on targeting CD8^+^ T cells metabolism as a potential strategy for cancer immunotherapy. Drugs like statins and avasimibe are effective and safe in managing lipid metabolism disorders, it’s worthwhile to investigate their potentials in targeting T cell lipid metabolism to generate therapeutic effects. In addition, combinatorial strategies like MEKi plus PD-L1 blockade or ACT seems promising to induce durable therapeutic effects by acting on the malignant cells and tumor-reactive CD8^+^ TILs. The interactions between cellular metabolism and epigenetics reinforce the importance of the immune metabolic microenvironment in the regulation of spatiotemporal gene expressions. Lipids and lipid metabolism are likely to induce the memory plasticity loss of CD8^+^ TILs. Enhancing CD8^+^ TILs cellular lipid metabolic fitness to generate memory cells or directly targeting memory CD8^+^ TILs lipid metabolism appears to be the trend in inducing long-term efficacies for cancer immunotherapy in mice. One thing to keep in mind, lipid metabolism widely exists in the process of cellular biochemistry. How to specifically regulate the lipid metabolism of CD8^+^ TILs and affect other normal physiological functions as little as possible remains to be explored. For example, pan-cancer lipidomic and TILs profiling will give us more precise view on the interaction between TME lipids and antitumor immunity for more selective targeted therapy. Surely, more in-depth studies of T cell lipids and lipid metabolism programs at the level of cellular signal transduction and gene expression regulation are still needed to help accelerating the clinical translations.

## Author contributions

YC wrote the manuscript. DW revised the manuscript. All authors contributed to the article and approved the submitted version.

## Funding

This study was supported by the National Natural Science Foundation of China (No.81771672), “2021 Shanghai Science and Technology Innovation Action Plan-Medical Innovation Research Special Project” (No.21Y11902000), “Cross Key Project of Mathematics and Medical Health” of the National Natural Science Foundation of China (No.12026608), Open fund project of Shenzhen BGI Institute of Life Science (No.BGIRSZ20200004) and Special Fund for Clinical Research of Zhongshan Hospital, Fudan University (No.2020ZSLC07), Key Subject Construction Program of Shanghai Health Administrative Authority (ZK2019B30), and Shanghai Engineering Research Center of Tumor Multi-Target Gene Diagnosis (20DZ2254300). The funding agencies had no role in the preparation.

## Conflict of interest

The authors declare that the research was conducted in the absence of any commercial or financial relationships that could be construed as a potential conflict of interest.

## Publisher’s note

All claims expressed in this article are solely those of the authors and do not necessarily represent those of their affiliated organizations, or those of the publisher, the editors and the reviewers. Any product that may be evaluated in this article, or claim that may be made by its manufacturer, is not guaranteed or endorsed by the publisher.
